# Supplementary material for: Discovery of Cell Number-Interstitial Fluid Volume (CIF) Ratio Reveals Secretory Autophagy Pathway to Supply eHsp90α for Wound Healing
Source: Cells. 2024 Jul 30;13(15):1280. doi: 10.3390/cells13151280 (PMC11312289; doi:10.3390/cells13151280)
Supplement: Supplementary file 1 [file cells-13-01280-s001.zip › Supplemental Materials-final.pptx]

## Slide 1
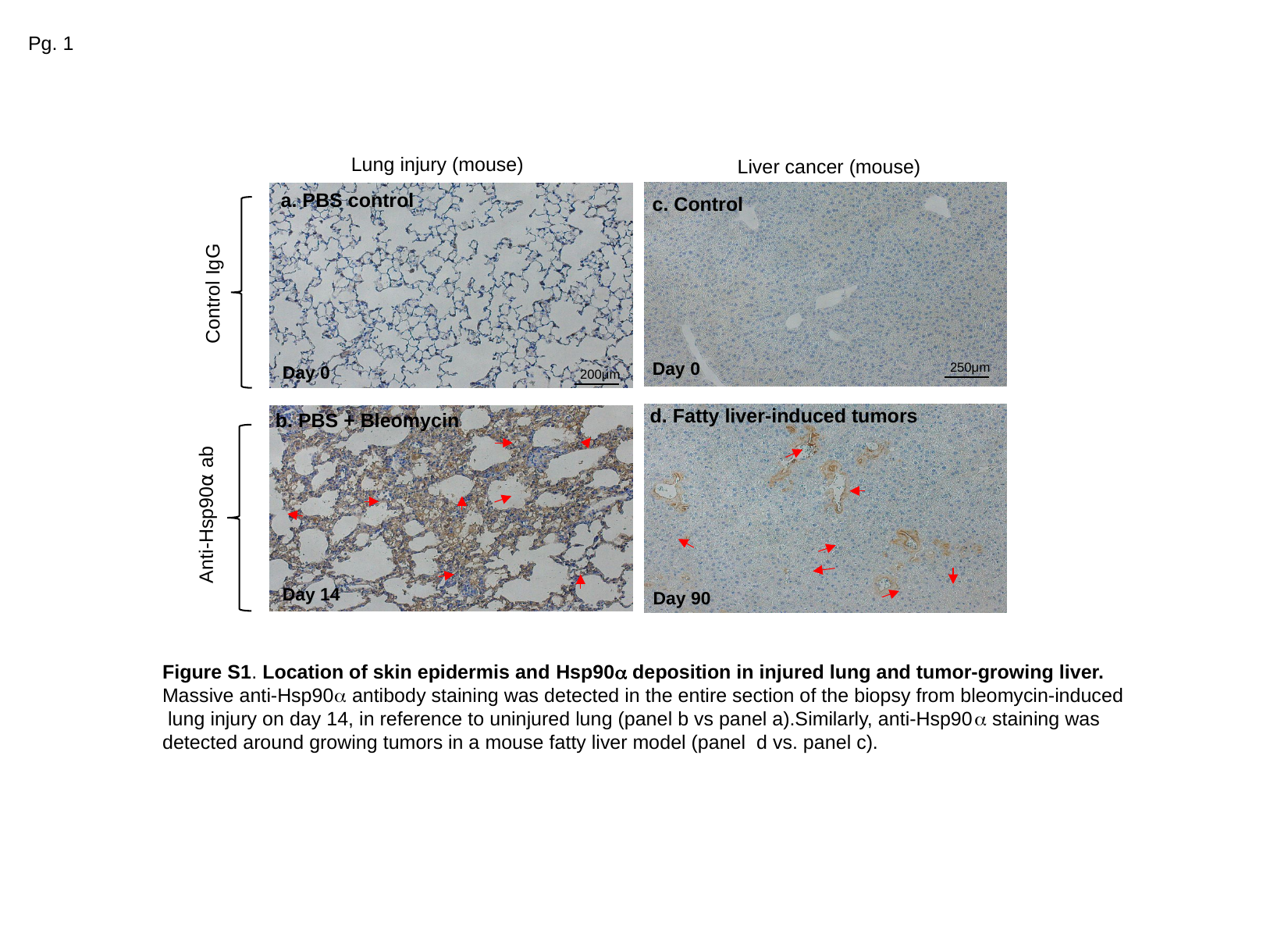

Pg. 1
Lung injury (mouse)
Liver cancer (mouse)
a. PBS control
c. Control
Control IgG
Day 0
250μm
Day 0
200μm
d. Fatty liver-induced tumors
b. PBS + Bleomycin
Anti-Hsp90⍺ ab
Day 14
Day 90
Figure S1. Location of skin epidermis and Hsp90α deposition in injured lung and tumor-growing liver.
Massive anti-Hsp90α antibody staining was detected in the entire section of the biopsy from bleomycin-induced
 lung injury on day 14, in reference to uninjured lung (panel b vs panel a).Similarly, anti-Hsp90α staining was
detected around growing tumors in a mouse fatty liver model (panel d vs. panel c).

## Slide 2
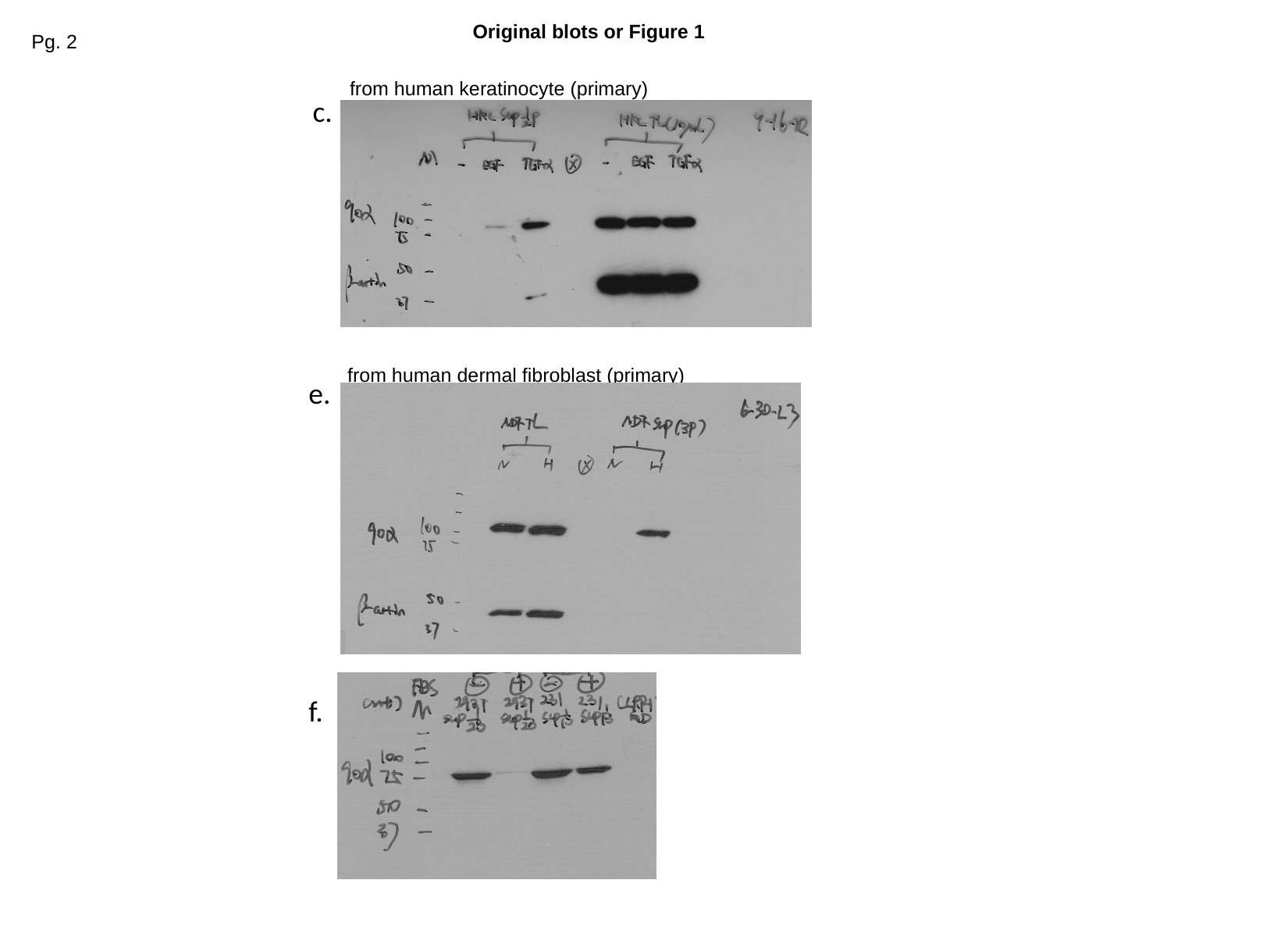

Original blots or Figure 1
Pg. 2
from human keratinocyte (primary)
c.
from human dermal fibroblast (primary)
e.
from human 293T
f.

## Slide 3
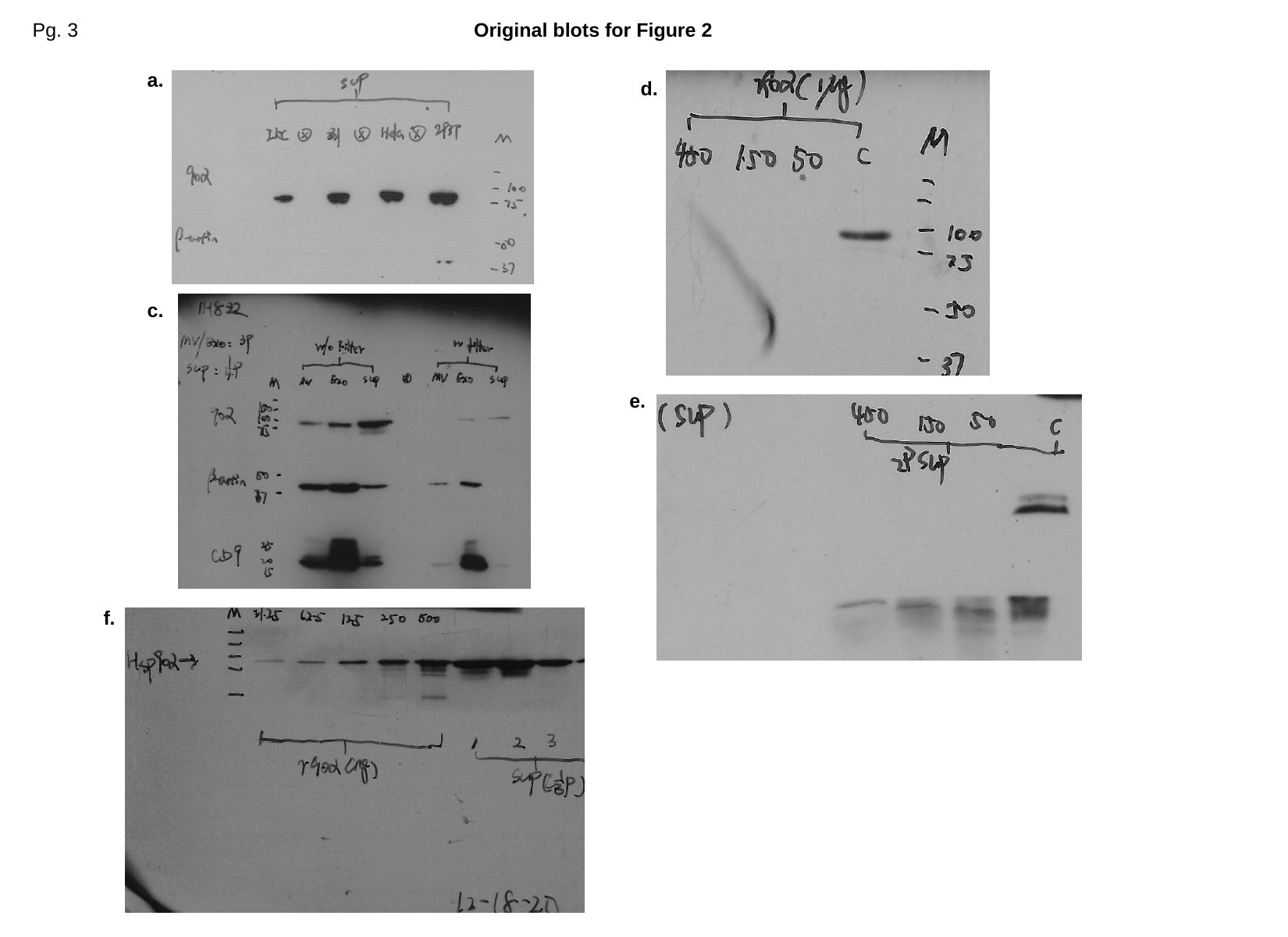

Pg. 3
 Original blots for Figure 2
a.
d.
c.
e.
f.

## Slide 4
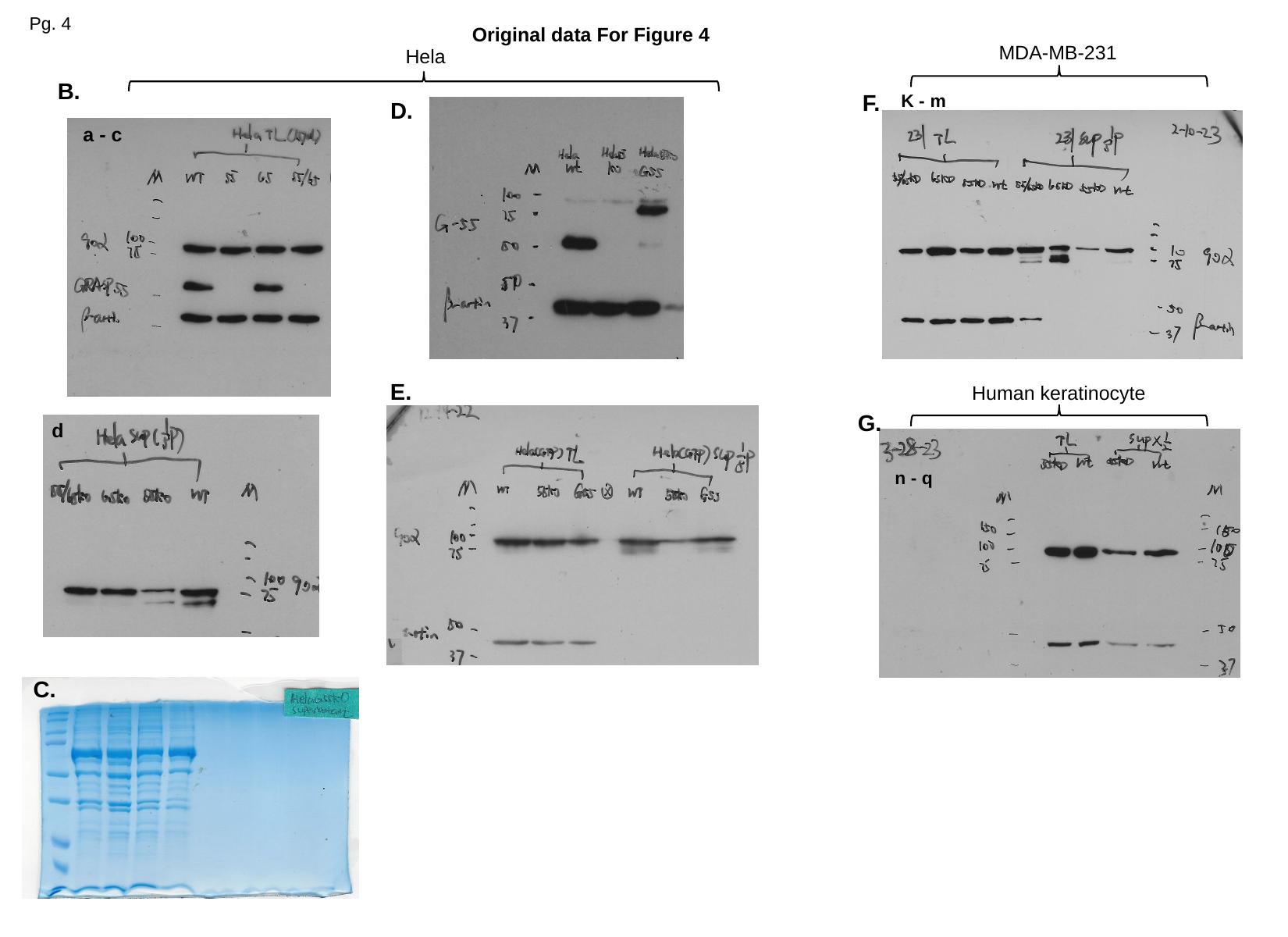

Pg. 4
Original data For Figure 4
MDA-MB-231
Hela
B.
F.
K - m
D.
a - c
E.
Human keratinocyte
G.
d
n - q
C.

## Slide 5
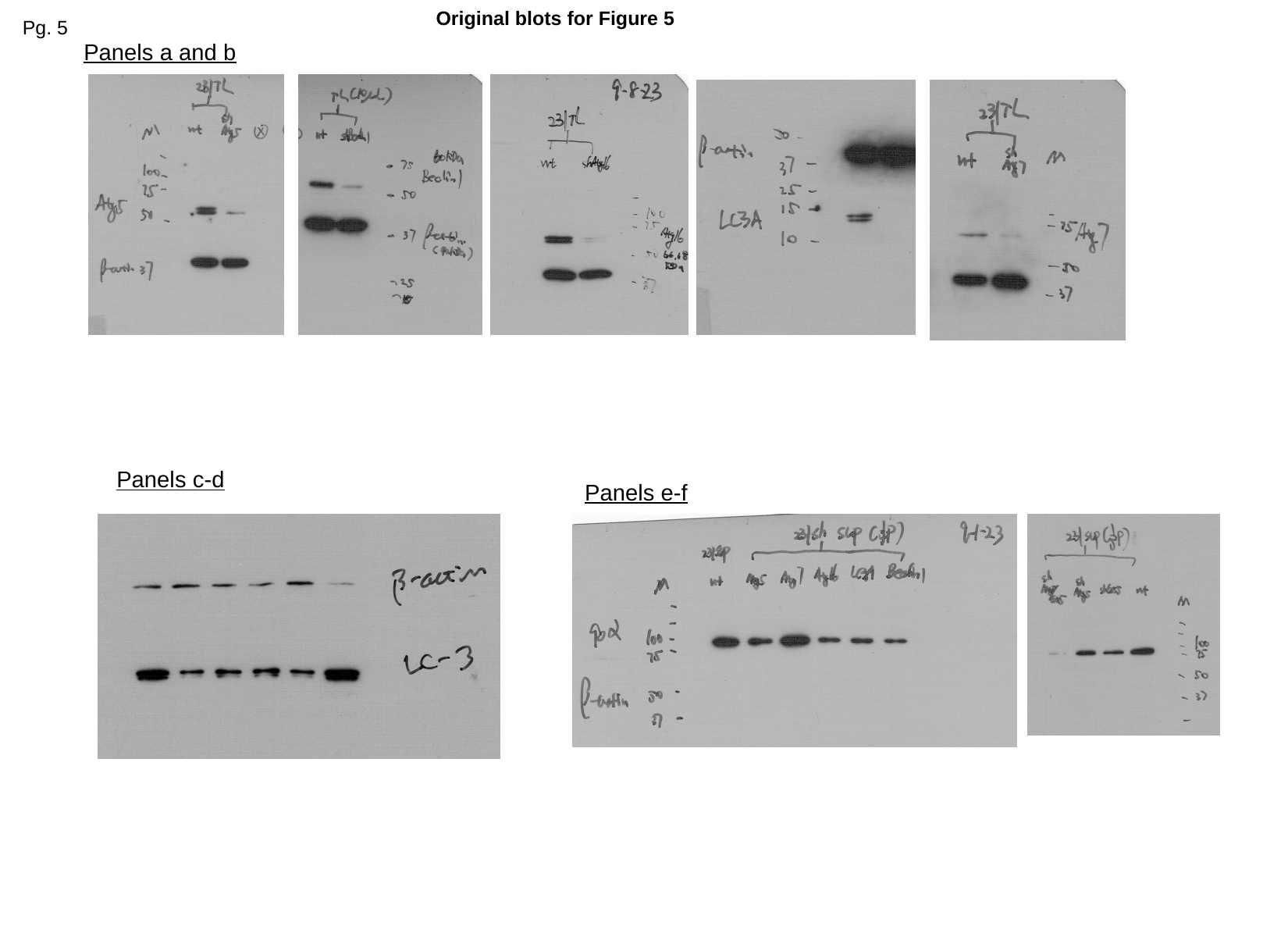

Original blots for Figure 5
Pg. 5
Panels a and b
Panels c-d
Panels e-f

## Slide 6
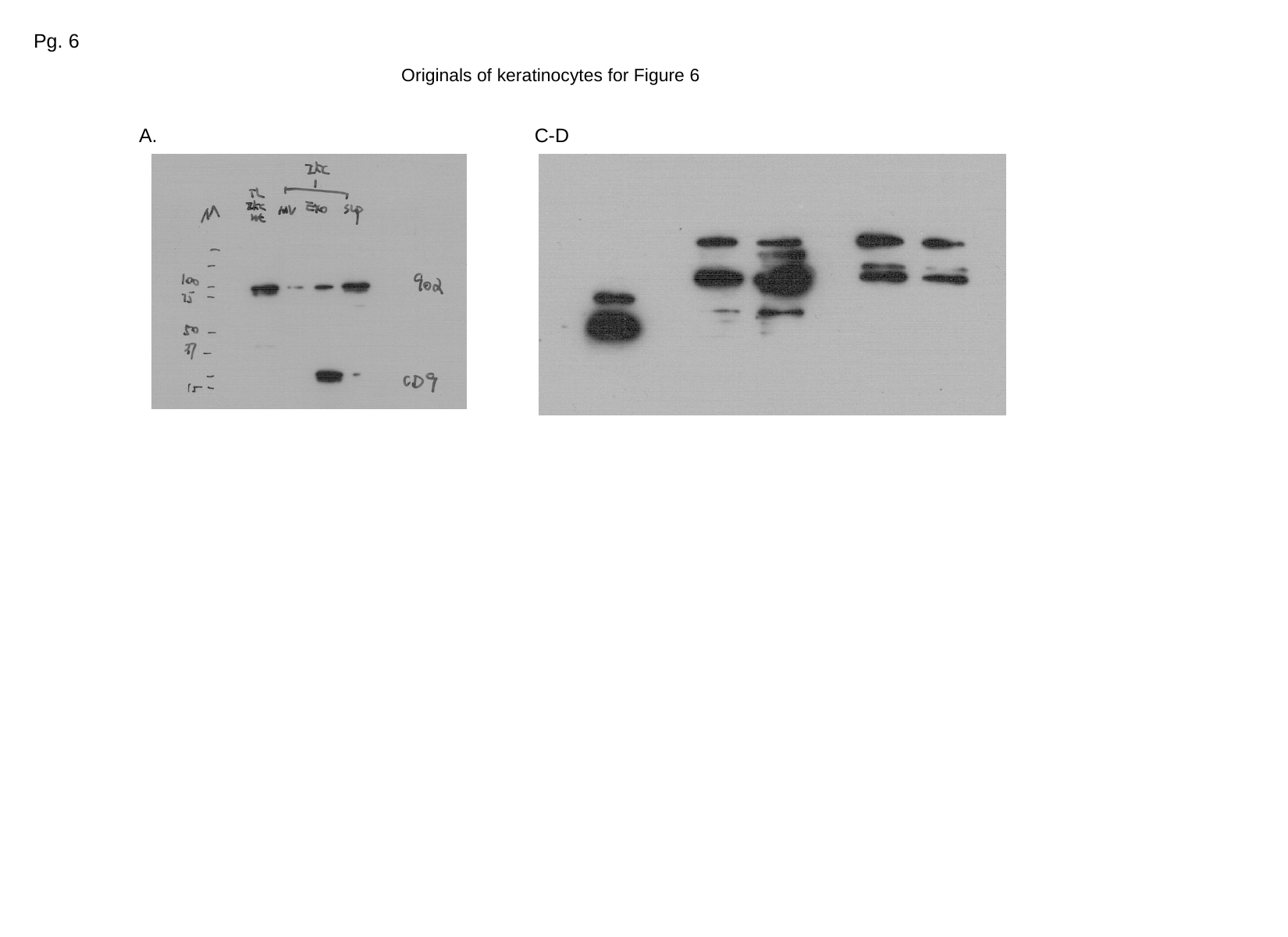

Pg. 6
Originals of keratinocytes for Figure 6
A.
C-D

## Slide 7
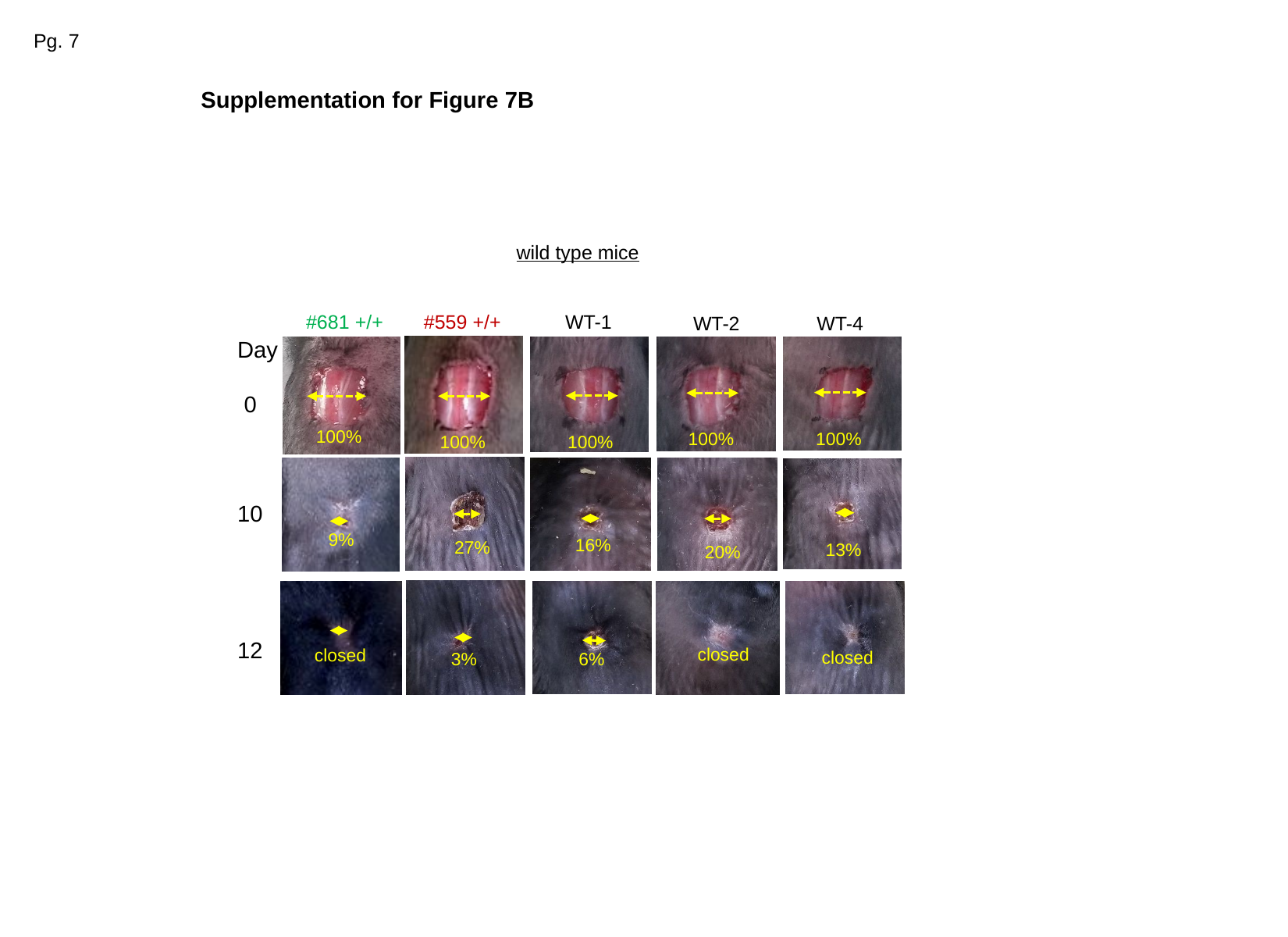

Pg. 7
Supplementation for Figure 7B
wild type mice
#681 +/+
#559 +/+
WT-1
WT-4
WT-2
Day
 0
10
12
100%
100%
100%
100%
100%
9%
16%
27%
13%
20%
closed
closed
closed
6%
3%

## Slide 8
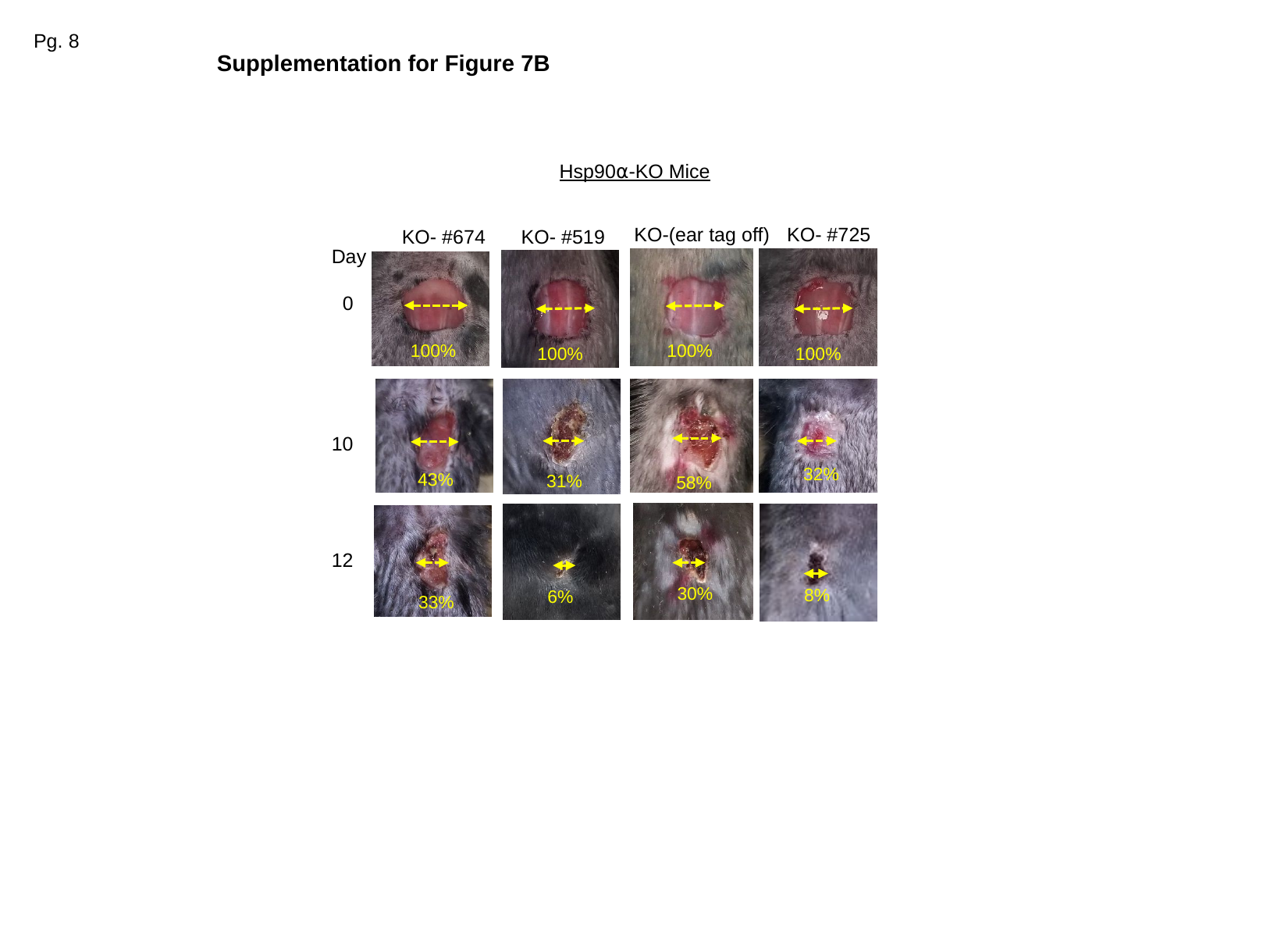

Pg. 8
Supplementation for Figure 7B
Hsp90⍺-KO Mice
KO- #725
KO-(ear tag off)
KO- #674
KO- #519
Day
 0
10
12
100%
100%
100%
100%
32%
43%
31%
58%
30%
8%
6%
33%

## Slide 9
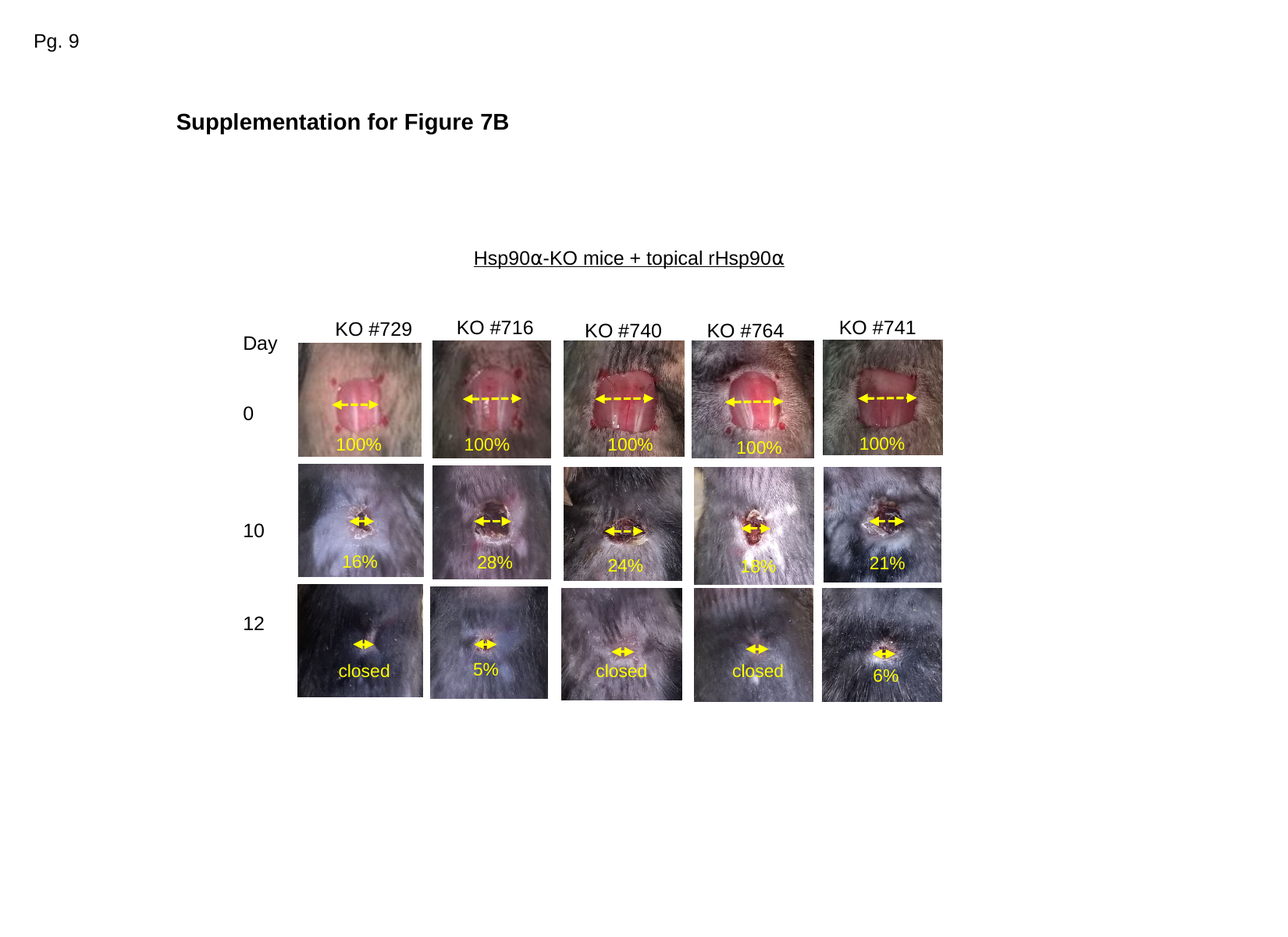

Pg. 9
Supplementation for Figure 7B
Hsp90⍺-KO mice + topical rHsp90⍺
KO #741
KO #716
KO #729
KO #740
KO #764
Day
0
10
12
100%
100%
100%
100%
100%
16%
28%
21%
24%
18%
5%
closed
closed
closed
6%

## Slide 10
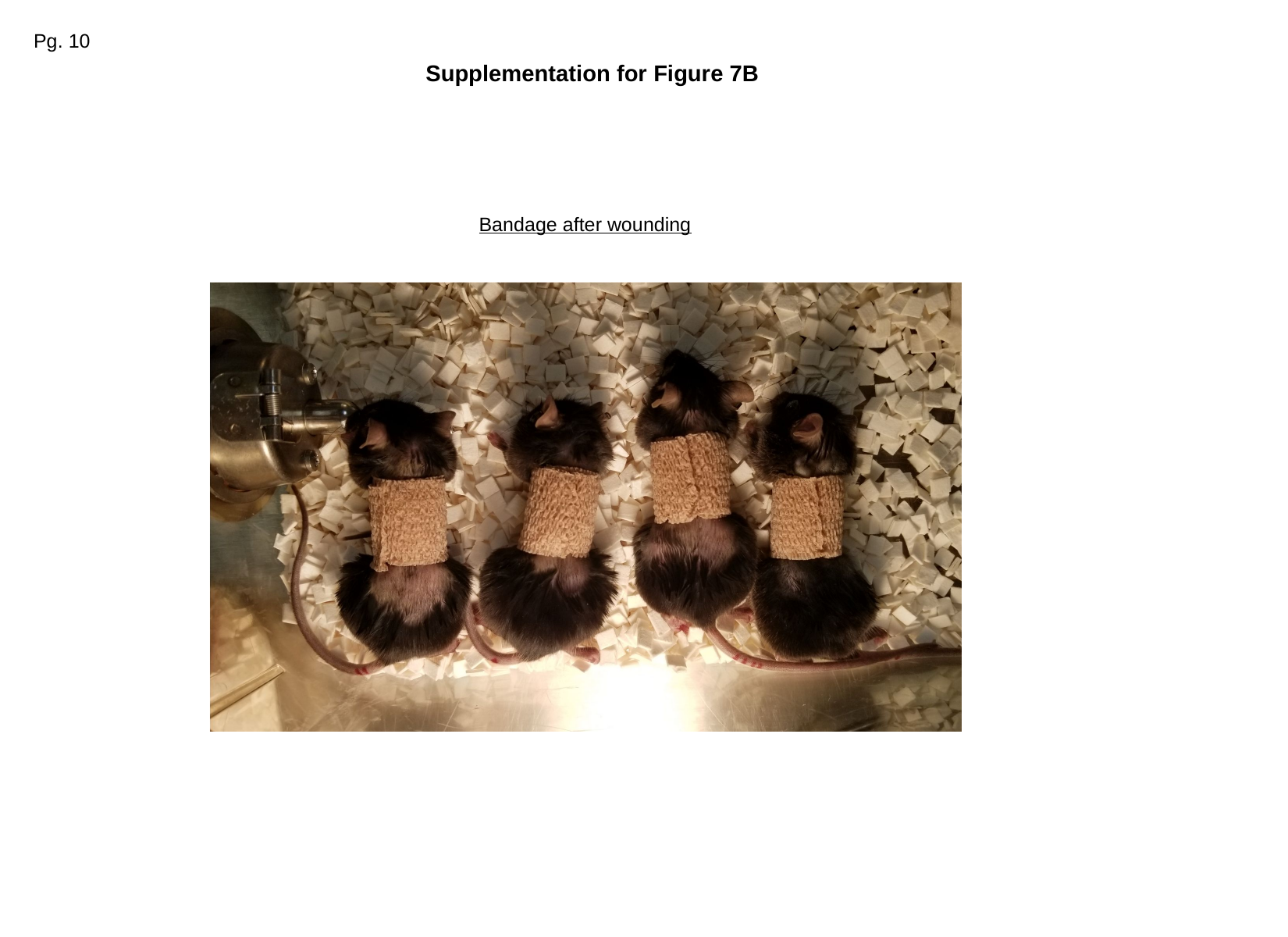

Pg. 10
Supplementation for Figure 7B
Bandage after wounding
